# Supplementary material for: Multi‐dataset identification of innovative feature genes and molecular mechanisms in keratoconus
Source: J Cell Mol Med. 2024 Sep 19;28(18):e70079. doi: 10.1111/jcmm.70079 (PMC11412914; doi:10.1111/jcmm.70079)
Supplement: Supplementary file 2 — Material S2. [file JCMM-28-e70079-s002.zip › 7.GSEA analysis/3.DBNDD1/KEGG_VASCULAR_SMOOTH_MUSCLE_CONTRACTION.html]

Details for gene set KEGG\_VASCULAR\_SMOOTH\_MUSCLE\_CONTRACTION[GSEA]

|  || Dataset | DBNDD1.group.cls#h\_versus\_l |
| Phenotype | group.cls#h\_versus\_l |
| Upregulated in class | l |
| GeneSet | KEGG\_VASCULAR\_SMOOTH\_MUSCLE\_CONTRACTION |
| Enrichment Score (ES) | -0.35791123 |
| Normalized Enrichment Score (NES) | -1.2691303 |
| Nominal p-value | 0.15090543 |
| FDR q-value | 0.6697562 |
| FWER p-Value | 0.974 |
Table: GSEA Results Summary

  

Fig 1: Enrichment plot: KEGG\_VASCULAR\_SMOOTH\_MUSCLE\_CONTRACTION      
 Profile of the Running ES Score & Positions of GeneSet Members on the Rank Ordered List

  

| SYMBOL | TITLE | RANK IN GENE LIST | RANK METRIC SCORE | RUNNING ES | CORE ENRICHMENT || 1 | ADRA1D | na | 1280 | 0.355 | -0.0075 | No |
| 2 | CACNA1F | na | 1766 | 0.316 | 0.0046 | No |
| 3 | CALML6 | na | 4335 | 0.202 | -0.0528 | No |
| 4 | KCNMB2 | na | 5953 | 0.152 | -0.0869 | No |
| 5 | PRKCG | na | 6020 | 0.150 | -0.0764 | No |
| 6 | ADCY7 | na | 6033 | 0.150 | -0.0643 | No |
| 7 | CALCRL | na | 6350 | 0.141 | -0.0617 | No |
| 8 | ADCY9 | na | 6468 | 0.137 | -0.0537 | No |
| 9 | PRKCQ | na | 6488 | 0.137 | -0.0429 | No |
| 10 | ADCY6 | na | 6632 | 0.133 | -0.0360 | No |
| 11 | PLA2G2F | na | 6705 | 0.131 | -0.0272 | No |
| 12 | PLA2G10 | na | 7003 | 0.124 | -0.0254 | No |
| 13 | MYH11 | na | 7776 | 0.108 | -0.0388 | No |
| 14 | CALML5 | na | 7930 | 0.105 | -0.0345 | No |
| 15 | PLA2G2D | na | 8134 | 0.101 | -0.0320 | No |
| 16 | NPR2 | na | 8325 | 0.098 | -0.0294 | No |
| 17 | ADCY4 | na | 8335 | 0.097 | -0.0215 | No |
| 18 | ADCY5 | na | 8470 | 0.095 | -0.0175 | No |
| 19 | PLCB2 | na | 9009 | 0.086 | -0.0259 | No |
| 20 | PLA2G12B | na | 9010 | 0.085 | -0.0188 | No |
| 21 | NPR1 | na | 9229 | 0.082 | -0.0183 | No |
| 22 | MAPK3 | na | 9865 | 0.073 | -0.0306 | No |
| 23 | CALML3 | na | 10231 | 0.068 | -0.0355 | No |
| 24 | KCNMB1 | na | 10268 | 0.068 | -0.0309 | No |
| 25 | PTGIR | na | 10527 | 0.064 | -0.0330 | No |
| 26 | ITPR2 | na | 10876 | 0.060 | -0.0382 | No |
| 27 | MAP2K2 | na | 10931 | 0.059 | -0.0348 | No |
| 28 | CALM2 | na | 11915 | 0.048 | -0.0592 | No |
| 29 | ROCK1 | na | 11947 | 0.048 | -0.0562 | No |
| 30 | RAMP2 | na | 12086 | 0.046 | -0.0563 | No |
| 31 | RAMP3 | na | 12483 | 0.043 | -0.0642 | No |
| 32 | PPP1CA | na | 12524 | 0.042 | -0.0619 | No |
| 33 | PRKCH | na | 12623 | 0.042 | -0.0613 | No |
| 34 | PPP1R14A | na | 12939 | 0.039 | -0.0672 | No |
| 35 | CACNA1D | na | 13311 | 0.036 | -0.0749 | No |
| 36 | PLA2G6 | na | 14048 | 0.030 | -0.0937 | No |
| 37 | MYL9 | na | 14830 | 0.024 | -0.1143 | No |
| 38 | BRAF | na | 15471 | 0.019 | -0.1312 | No |
| 39 | PLA2G4A | na | 15676 | 0.018 | -0.1356 | No |
| 40 | PRKX | na | 15799 | 0.017 | -0.1376 | No |
| 41 | CALM1 | na | 15870 | 0.017 | -0.1383 | No |
| 42 | ITPR3 | na | 16903 | 0.010 | -0.1673 | No |
| 43 | MYL6 | na | 17272 | 0.008 | -0.1773 | No |
| 44 | PLA2G2E | na | 17656 | 0.005 | -0.1879 | No |
| 45 | MYL6B | na | 17881 | 0.004 | -0.1941 | No |
| 46 | PPP1CB | na | 17957 | 0.003 | -0.1960 | No |
| 47 | GNA11 | na | 17984 | 0.003 | -0.1965 | No |
| 48 | MYLK2 | na | 17990 | 0.003 | -0.1964 | No |
| 49 | PLA2G12A | na | 18106 | 0.002 | -0.1996 | No |
| 50 | RAF1 | na | 19005 | -0.005 | -0.2251 | No |
| 51 | RHOA | na | 19096 | -0.005 | -0.2273 | No |
| 52 | ADCY2 | na | 19167 | -0.006 | -0.2288 | No |
| 53 | ITPR1 | na | 19252 | -0.006 | -0.2307 | No |
| 54 | PLA2G4E | na | 19468 | -0.008 | -0.2362 | No |
| 55 | PLCB3 | na | 19605 | -0.010 | -0.2394 | No |
| 56 | PLCB4 | na | 19707 | -0.010 | -0.2414 | No |
| 57 | GNA12 | na | 20454 | -0.017 | -0.2616 | No |
| 58 | CALM3 | na | 20684 | -0.019 | -0.2666 | No |
| 59 | PRKCD | na | 20754 | -0.020 | -0.2670 | No |
| 60 | PPP1CC | na | 20793 | -0.020 | -0.2664 | No |
| 61 | PRKACA | na | 20814 | -0.020 | -0.2653 | No |
| 62 | ACTA2 | na | 20830 | -0.021 | -0.2640 | No |
| 63 | ROCK2 | na | 21182 | -0.024 | -0.2722 | No |
| 64 | PRKCA | na | 21412 | -0.026 | -0.2767 | No |
| 65 | GNAQ | na | 21825 | -0.030 | -0.2860 | No |
| 66 | GNAS | na | 22042 | -0.033 | -0.2896 | No |
| 67 | PPP1R12B | na | 22296 | -0.035 | -0.2940 | No |
| 68 | PLA2G2C | na | 22365 | -0.036 | -0.2930 | No |
| 69 | MAPK1 | na | 22705 | -0.040 | -0.2995 | No |
| 70 | ADCY8 | na | 23069 | -0.044 | -0.3063 | No |
| 71 | JMJD7-PLA2G4B | na | 23109 | -0.044 | -0.3038 | No |
| 72 | CACNA1C | na | 23124 | -0.045 | -0.3005 | No |
| 73 | ADCY3 | na | 23170 | -0.045 | -0.2980 | No |
| 74 | ARHGEF11 | na | 23636 | -0.052 | -0.3071 | No |
| 75 | CALD1 | na | 23773 | -0.053 | -0.3067 | No |
| 76 | PPP1R12A | na | 24009 | -0.056 | -0.3088 | No |
| 77 | ARHGEF12 | na | 24511 | -0.063 | -0.3180 | No |
| 78 | ARAF | na | 24817 | -0.068 | -0.3211 | No |
| 79 | KCNMA1 | na | 25759 | -0.083 | -0.3414 | No |
| 80 | ADORA2B | na | 26330 | -0.094 | -0.3501 | Yes |
| 81 | GUCY1A2 | na | 26540 | -0.098 | -0.3480 | Yes |
| 82 | PRKACB | na | 26730 | -0.102 | -0.3450 | Yes |
| 83 | ADORA2A | na | 26738 | -0.102 | -0.3367 | Yes |
| 84 | GNA13 | na | 26859 | -0.104 | -0.3315 | Yes |
| 85 | ADRA1A | na | 27600 | -0.121 | -0.3428 | Yes |
| 86 | AVPR1B | na | 27779 | -0.126 | -0.3375 | Yes |
| 87 | RAMP1 | na | 27807 | -0.126 | -0.3278 | Yes |
| 88 | PRKACG | na | 28125 | -0.134 | -0.3258 | Yes |
| 89 | MYLK3 | na | 28605 | -0.146 | -0.3275 | Yes |
| 90 | PLA2G3 | na | 28607 | -0.146 | -0.3154 | Yes |
| 91 | MYLK | na | 28743 | -0.150 | -0.3069 | Yes |
| 92 | PLA2G5 | na | 29074 | -0.159 | -0.3032 | Yes |
| 93 | MAP2K1 | na | 29246 | -0.164 | -0.2945 | Yes |
| 94 | CYP4A11 | na | 29497 | -0.172 | -0.2875 | Yes |
| 95 | ADCY1 | na | 29881 | -0.185 | -0.2832 | Yes |
| 96 | KCNMB3 | na | 30084 | -0.191 | -0.2732 | Yes |
| 97 | ARHGEF1 | na | 30268 | -0.196 | -0.2622 | Yes |
| 98 | ACTG2 | na | 30404 | -0.201 | -0.2494 | Yes |
| 99 | AVPR1A | na | 30657 | -0.209 | -0.2393 | Yes |
| 100 | PRKG1 | na | 31202 | -0.229 | -0.2360 | Yes |
| 101 | AGTR1 | na | 31373 | -0.235 | -0.2214 | Yes |
| 102 | PLA2G1B | na | 31847 | -0.256 | -0.2138 | Yes |
| 103 | PLCB1 | na | 32023 | -0.265 | -0.1969 | Yes |
| 104 | ADRA1B | na | 32178 | -0.275 | -0.1786 | Yes |
| 105 | PRKCE | na | 32256 | -0.280 | -0.1576 | Yes |
| 106 | CYP4A22 | na | 32907 | -0.321 | -0.1497 | Yes |
| 107 | KCNMB4 | na | 33958 | -0.424 | -0.1449 | Yes |
| 108 | PRKCB | na | 34153 | -0.452 | -0.1130 | Yes |
| 109 | CACNA1S | na | 34256 | -0.474 | -0.0766 | Yes |
| 110 | PLA2G2A | na | 34424 | -0.522 | -0.0381 | Yes |
| 111 | EDNRA | na | 34526 | -0.565 | 0.0058 | Yes |
Table: GSEA details [plain text format]

  

Fig 2: KEGG\_VASCULAR\_SMOOTH\_MUSCLE\_CONTRACTION      
 Blue-Pink O' Gram in the Space of the Analyzed GeneSet

  

Fig 3: KEGG\_VASCULAR\_SMOOTH\_MUSCLE\_CONTRACTION: Random ES distribution      
 Gene set null distribution of ES for **KEGG\_VASCULAR\_SMOOTH\_MUSCLE\_CONTRACTION**
